# Sustaining Forest Ecosystem Services Through Social Enterprises: Motivations and Challenges from a Case Study in Scotland

**DOI:** 10.1007/s11842-021-09484-7

**Published:** 2021-05-20

**Authors:** Shaohui Zhang, James S. Paterson, Teppo Hujala

**Affiliations:** 1 School of Forest Sciences, University of Eastern Finland, Joensuu, Finland; 2 School of GeoSciences, University of Edinburgh, Edinburgh, UK

**Keywords:** Forest ecosystem services, Social enterprises, Scotland, Thematic analysis

## Abstract

Both the concepts of ecosystem services and social enterprise have gained popularity as means of addressing social and environmental issues in recent years. But while research on ecosystem services has focused on non-governmental organisation and local community-led approaches, the role of social enterprises has received less attention. In Scotland, social enterprises play an important role in delivering social and environmental justice, as well as reaping economic benefits through forest-based practices. These practices are often small-scale and attract participants from local communities. But despite this prominence their motivations and the challenges they face remain largely unexplored. This research attempts to integrate both concepts mainly using field observations and interviews, and to reinforce the findings with the existing literature. It explores the motivations and challenges of social enterprises in sustaining forest ecosystem services through a case study, and discusses their potential within the context of current policy. The research suggests that social enterprises are equipped to tackle a range of social and environmental issues. The enterprise under investigation aims to improve participants’ wellbeing and employability, as well as to provide public education and promote environmental awareness. But these efforts are often hindered by the difficulties of high requirements of the manager and staff members in balancing multiple objectives and most importantly, of managing financial risks. The research concludes that a practical method of assessing cultural ecosystem services as well as the use of innovative funding norms may resolve these challenges, allowing social enterprises to fill the current policy gap and create synergies in the fields of ecosystem services and social enterprise.

## Introduction

### The Origin and Development of Ecosystem Services

The concept of ecosystem services has sparked widespread interest in recent years. Its core value lies in its emphasis on the interdependence and interconnectedness of human beings and ecosystems (Quine et al. 2011).

The most popular definition of ecosystem services arguably comes from the Millennium Ecosystem Assessment (MEA) which states that “ecosystem services are the benefits people obtain from ecosystems, including provisioning, regulating, cultural and supporting services” (MA 2005). It has been the key driver in raising awareness and acceptance of ecosystem services and has attracted great interest in the goods and services that nature contributes to human society and wellbeing (Chaudhary et al. 2015). As a result, the concept of ecosystem services has increased in popularity, particularly in policy and academic fields, and has witnessed rapid institutionalisation and operationalisation worldwide (Bouwma et al. 2018).

In Europe, the Common International Classification of Ecosystem Services (CICES) (Haines-Young and Potschin 2018)—a uniform definition of consistent ecosystem services categories—has been supported and developed by the European Environment Agency (EEA) to standardise the classification of ecosystem services since 2009. This standardisation was both necessary and important as it provided a systematic guidance on the naming and describing of ecosystem services in order to prevent further overlapping issues related to ecosystem services. Nevertheless, the intention is not to create a fixed classification as it is still evolving, but rather to develop a framework capable of accommodating data from different areas.

In the UK, ecosystem services has been included extensively in various government publications and policy announcements. The year 2007 marked the formal introduction of the ecosystem approach when the Department of Environment, Food and Rural Affairs (Defra) published its first action plan for embedding an ecosystems approach (Defra 2007). In 2009, the UK conducted systematic ecosystem assessments (including forests) and established itself as a pioneer of this approach. In 2011 the UK National Ecosystem Assessments (NEA) was published, which provided the first country-wide analysis and assessment of natural environments and their contribution to social and economic development (NEA 2011).

This ecosystem services thinking has been widely applied in the UK forest sector as an emerging paradigm in order to reconcile tensions resulting from limited forest resources. The fact that forests represent only 13% of the total land (Forestry Research 2019) may have exacerbated tensions between different interest groups (e.g., nature conservation vs. productive forestry). Hence, the concept of ecosystem services provides a holistic way of looking at forest ecosystems to ensure a healthy, resilient and sustainable environment for current and future generations.

### Social Enterprise Emerging in Scotland

In Scotland, social enterprise emerged in the late 1990s, yet the definition and meaning of the term “social enterprise” still remains a highly contested concept (Shaw and de Bruin 2013). Social enterprises are commonly defined as entrepreneurial activities that do not trade for profit but are rather established for a social or environmental purpose. As well as their social and environmental aims, social enterprises are also combined with a set of business-like financial and managerial systems to meet their commercial objectives that are needed to cover their operational costs (Carlo and Jacques 2001). Despite all efforts to frame the concept of social enterprise, there is yet no fixed definition that is able to cover all the dynamics of these organisations (Teasdale 2010).

To help recognise and identify social enterprises, a broad consensus nevertheless exists regarding the benchmark criteria and values on which social enterprises should be based. In 2016, the Scottish Government adopted the values and behaviours of social enterprises—namely the Voluntary Code of Practice for Social Enterprises in Scotland—established by the Scottish Social Enterprise Community, which describes social enterprise as follows:

A social enterprise is a trading business—selling goods and services—but whose primary objective is to achieve social and/or environmental benefit. Social enterprises are different from those charities and voluntary organisations which do not aspire to financial independence through trading. Regardless of its legal form, the constitution of a social enterprise will include the requirements that profits are invested in the business or the beneficiary community—and not distributed to private owners, shareholders or investors. (The Scottish Social Enterprise Community 2015).

Social enterprises can play an important role in tackling unique rural challenges faced by those rural regions where infrastructure and resources are inadequate (Ludvig et al. 2018). One particular characteristic of social enterprises in Scotland is their increasing connection with rural development. About a third of the nation’s social enterprises are located in rural Scotland and tend to be small in size and focused on solutions to rural challenges (Community Enterprise in Scotland 2017). In the case of forest-related social enterprises, they often conduct forest practices on a small scale and may not necessarily involve local communities (Ambrose-Oji, Lawrence and Stewart 2015).

Considering the diverse contexts in which social enterprises have been established and are thriving in Scotland, it seems likely that an understanding of the defining characteristics of social enterprise will continue to evolve. The definition used here is intended only as a point of reference for defining a social enterprise, rather than an attempt to join the definitional debate. Instead, this paper will concentrate on the interaction between social enterprises and ecosystem services.

### Research Objectives

While there has been growing interest in the non-governmental organisation (NGO) and community approaches of forest projects, social enterprises have not received the same degree of attention and prominence in policy (Ambrose-Oji et al. 2015). In addition, ecosystem services thinking seems to have been adopted by a wide range of stakeholders in order to justify and support a variety of activities and goals (Kullet al. 2015). Nevertheless, so far, these two concepts are often investigated in separate fields. Research focused on enabling forest ecosystem services through social enterprises is very limited, especially in developed countries (Macqueen 2008). Furthermore, it remains to be explored how social enterprises can help sustain forest ecosystem services and what are the challenges they face. By synthesising these the two popular concepts of social enterprise and forest ecosystem services, this paper attempts to fill the gaps in current knowledge. It seeks to improve the understanding of social enterprise’s ability to sustain forest ecosystem services by:

1. Exploring the current policy context influencing the governance of social enterprise over forest resources.
2. Identifying the motivations and key challenges for social enterprises in sustaining forest ecosystem services.

This paper uses a case study of the New Caledonian Woodlands from Scotland. Through small-scale forestry and forest-related practices, it aims to address mental health issues and to promote environmental sustainability by providing access to forest ecosystem services. It provides a real-world context within which the research objectives can be explicitly explored.

## Methodology

### Study Context

The subject of forest ecosystem services has been well addressed in Scottish forestry policy. The first Scottish Forestry Strategy set out the principle that Scottish forests should contribute to the wellbeing of the people through a rich forest culture (Forestry Commission Scotland 2000). Although people in the UK almost universally agree that forests are important to them, only 63% have themselves visited forested areas in the last few years (Forest Research 2017).

**Fig. 1:**
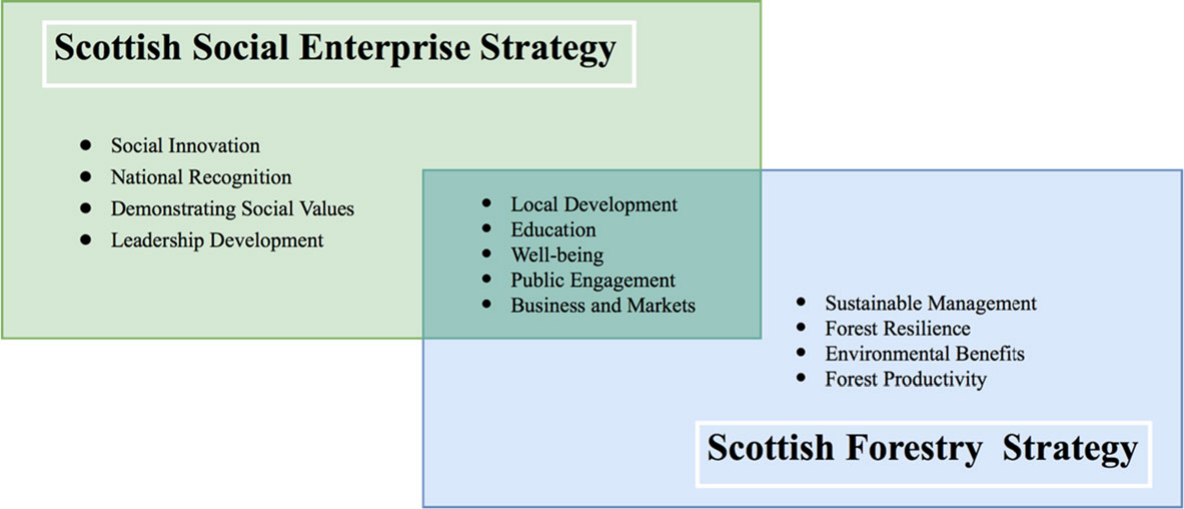
Key themes of Scottish social enterprise strategy and forestry strategy

Adapted from The Scottish Government (2016) and The Scottish Government (2019).

In addition, it is estimated that there are approximately 5600 social enterprises based across Scotland (Community Enterprise in Scotland 2017). Social enterprises have come to the forefront as a method of tackling social issues and of restructuring various public services (Sepulveda 2015). While social enterprises continue to grow in number and scale, they are not yet significantly involved in certain areas, including in forest ecosystem services. This suggests that social enterprises have an important role to play in addressing people’s unsatisfied needs through forest-related practices. Figure 1 illustrates some of the main themes that are listed in Scotland’s Social Enterprise Strategy and Forestry Strategy. The overlapping areas indicate the opportunity for forest-related social enterprises to accommodate both policies and create synergies.

Despite the claim that Scotland is “the most supportive environment in the world for social enterprise” (Roy et al. 2015), existing literature has recorded some commonly-seen challenges faced by social enterprises in the country. Some challenges derive from ambiguities in the definition of social enterprise (Hazenberg et al. 2016), its legal form (Hare et al. 2007) and policy direction (Farmer et al. 2008). These conceptual factors fundamentally shape the social enterprise discourse, which when misunderstood or misinterpreted, often cause challenging tensions among internal and external stakeholders. Others include failure to gain recognition from authorities (Henderson et al. 2019), difficulties of evaluating values added by social enterprises (Hartley et al. 2019) and distinct barriers for social enterprises especially in rural settings (Steinerowski and Steinerowska-Streb 2012). Most of these challenges are reported by social enterprises when delivering social benefits related to health and social care, employability and local development, as these are the areas on which social enterprises in Scotland are predominantly focused. This paper investigates a social enterprise that delivers not only social but also environmental benefits derived from forest ecosystems and, in doing so, hopes to offer new insights into the topics of both social enterprise and ecosystem services.

### Case Study: New Caledonian Woodlands

Established in 2006, New Caledonian Woodlands was located in the outskirts of Edinburgh and aimed to deliver social and environmental benefits to people from local and nearby council areas through forest-related practices. It was a small-scale social enterprise with one manager and six staff members. The social enterprise was not community-owned and the staff members were not necessarily of local origin, a characteristic which may distinguish it from community-led projects. The forests, where the social enterprise conducts its activities, were planted under the Millennium Forest Project in and around Edinburgh; and the City of Edinburgh Council is responsible for forest management. Yet, the forests were subcontracted to New Caledonian Woodlands and consequently are directly managed by the social enterprise.

The social enterprise aimed to improve people’s mental wellbeing and to encourage environmental sustainability through forest-related activities. In order to achieve this, it created two kinds of project: ‘people’ project and ‘planet’ project, with each having 3–4 subordinate programmes targeted at their respective aims. The ‘people’ project focused on reducing social isolation and improving mental wellbeing through collective forest-based activities, such as thinning, planting trees and collecting fuelwood. The ‘planet’ project aimed to improve awareness of sustainability and to guide participants towards a greener lifestyle by improving their environmental awareness and education through lessons and leisure activities in the forest. These projects thus could be seen as a medium that bridged forests (ecosystem servicers provider) and society (ecosystem services receiver) and they were expected to sustain forest ecosystem services.

New Caledonian Woodlands worked with a maximum of over 1000 people every year. Participants in the ‘people’ project often experienced various degrees of mental health issues and were usually referred by mental health practitioners, but the ‘planet’ project was available to the wider public. At the time of this research, the social enterprise was experiencing financial difficulties, and it was later forced to close at the end of 2018 as a consequence. Nevertheless, the legacy of the social enterprise has been continued by former practitioners until now. Similar activities are occasionally resumed, and they are expected to exert a significant influence on people’s wellbeing during and after the COVID-19 pandemic.

All of this makes New Caledonian Woodlands an especially useful case study of the challenges experienced by forest-based social enterprises. It also sheds light on how social enterprise can continue to spread its social and environmental benefits even if forced to close by unexpected events.

### Overview of the Research Methods

The research method adopted in this paper is a combination of semi-structured in-depth interviews, field observations, and literature review. Semi-structured in-depth interviews reveal much about the social and cultural practices experienced by the respondents (Gubrium and Holstein 2001). It helps to provide the research context and to expose knowledge that is perceived differently by individuals involved in the social enterprise. Field observation and photography are proposed as being “especially effective in generating evidence that other methods—especially interviews—cannot” (Rose 2014, p. 28), and thus were also used to complement interview material. It is believed that these methods helped the researcher collect sufficient data to represent the structure of the social enterprise and the effectiveness of its activities.

**Fig. 2:**
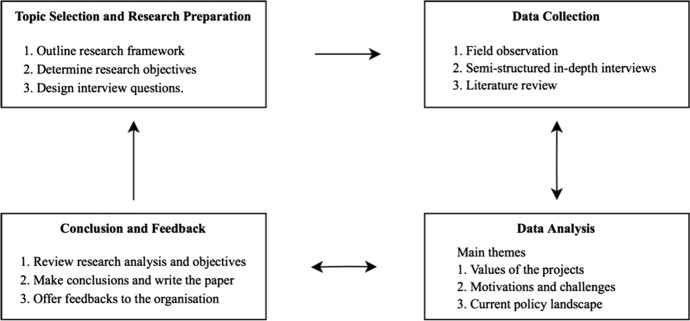
Framework of the methodology used in this paper. Adapted from Miles and Hubeman (1994). *Note* The arrows indicate the analytical process, sometimes linear and often iterative and recursive. For example, more tentative themes were identified in the initial data analysis. However, it was felt that some themes lacked solid data support and thus were abandoned before a conclusion was attempted. A literature review was simultaneously in process in order to measure the latest findings against previous experience.

In addition, findings from reviewing the literature were linked with, and validated against, discoveries from interviews and fieldwork. Research in the literature drew from a wide range of papers, with the focus on three domains: ecosystem services, social enterprise, and related UK and Scottish policies. Research data collected from these multiple sources are expected to provide a solid understanding of the phenomena under investigation. Figure 2 is included to illustrate the methodological framework adopted in this paper.

### Data Collection and Analysis

The researcher joined practitioners from both the ‘people’ and ‘planet’ projects and undertook field observations (n = 4) from April to June 2018. The observation events, each of which lasted a full day, covered the three programmes of the ‘people’ project and one programme of the ‘planet’ project according to their availability. This research complied with the Research Ethics and Integrity framework approved by the University of Edinburgh and these guidelines were fully taken into consideration due to the sensitivity surrounding the participants. During the events, the researcher conducted informal interviews with several participants (n = 7) to assess their general satisfaction with and feedbacks on the programmes, with significant comments noted down by hand. The researcher also took photographs, mainly images representing the working environment and outcomes of the programmes (such as activities in the forest and forest products made by the participants). Although data obtained from field observations and photographs were not used directly in the results, it is believed that through observation and participation, a positive rapport was built up between the researcher and the individuals who were the subjects of this case study. It was hoped that, in this way, the establishment of trust and the creation of comfort before interviewing the practitioners could improve the data quality and subsequently lead to a more precise interpretation of these data.

In addition, seven semi-structured in-depth interviews, each lasting 30–40 min, with New Caledonian Woodlands employees were conducted in person and audio recorded with their prior consent. Given that the research topic involves well-being, both physical and mental health, this interview format was chosen as it has been recorded as being particularly useful for understanding social issues relevant to health care settings (DiCicco-Bloom and Crabtree 2006). One additional set of interview questions was answered by the former manager via e-mail due to distance, as it was felt that the historical development of the social enterprise should not be neglected. The interviewees selected covered all levels and functions of the social enterprise in question, from managers to individual practitioners, in order to provide a holistic picture of the social enterprise and its projects. Table 1 provides a summary with examples of the multiple data sources.

Data analysis followed the thematic analysis approach (Braun and Clarke 2006). Discoveries from interviews and fieldworks (primary data) were first categorised, synthesised and thematically analysed within individual main themes first; and then compared with, and validated against with evidence from existing literature (secondary data). The main themes included: (1) the values of social enterprise projects; (2) the motivations behind, and the challenges faced by social enterprises; (3) the current policy landscape regarding ecosystem services and social enterprises. It is believed that this thematic analysis approach can present the social practices experienced by research participants in a “transparent and credible” way (Guest, MacQueen and Namey 2012, p. 15).

**Table 1:** Summary and examples of the data

Data sources	Sample size	Main themes	Examples
Semi-structured in-depth interviews with staff member	7 people	Values of social enterprise projects	How are the social, environmental and economic benefits are delivered by your organisation?
		Motivations and challenges	What motivates social enterprises to promote forest ecosystem services?
		Current policies regarding forest social enterprise	How do you think the current UK/Scottish Government policy contributes to, or hinders, the development of forest social enterprise?
Informal interviews with participants	7 people	Forest ecosystem services	How do you like working in the forest?
		Social enterprises	What do you think of the projects carried out by New Caledonian Woodlands?
Field observations and photography	4 visits	‘People’ project (3 visits)	Participants crafting wooden products (targeting wellbeing)
		‘Planet’ project (1 visit)	Participants thinning woodlands under guidance (targeting sustainability)

## Results

The research revealed that the projects conducted by the social enterprise exploited certain forest ecosystem services and transformed their benefits into different forms before bringing them to the participants and society. The main forest ecosystem services of which the social enterprise aims to make full use through their projects are provisioning, regulating and cultural ecosystem services.

To give some concrete examples, the ‘people’ project involves collecting non-timber forest products (provisioning services) such as berries and small woods, and subsequently crafting them to organic products and household firewood. The ‘planet’ project involves practising woodland management skills such as planting new trees, pruning and thinning (regulating services) to encourage biodiversity and woodland culture. In addition, both projects regularly arrange some physical activities, such as leisure walks, and casual lessons of environmental education within the amenity provided by forests (cultural services). Notably, the supporting services is also concerned given the trees planted and managed, although they are not regarded as a priority by the social enterprise.

**Fig. 3:**
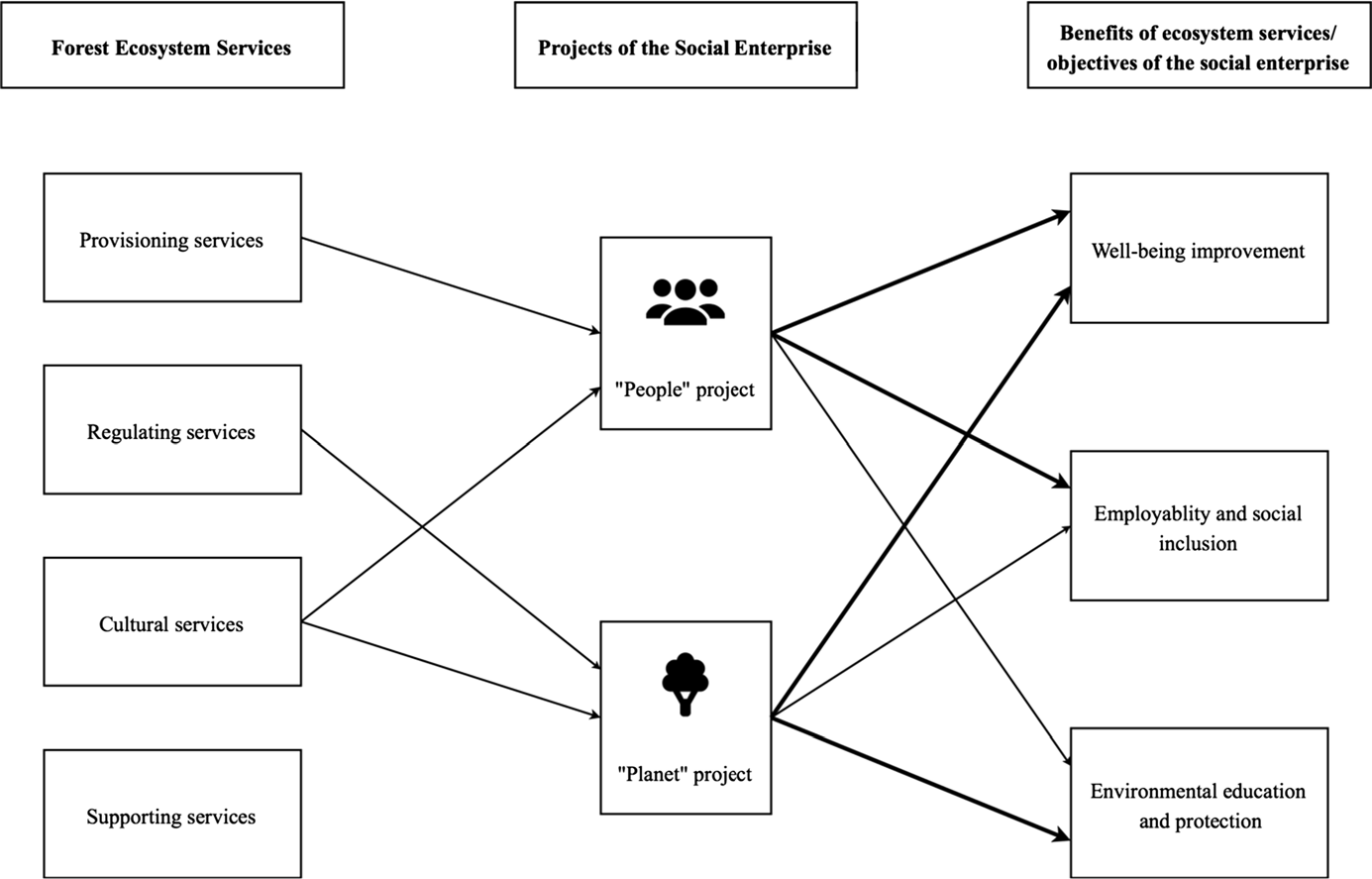
Linkages between the social enterprise and forest ecosystem services involved. The widths of the arrow lines indicate the relative priority given to the project objectives

By carrying out these activities, the expected benefits include improving wellbeing, strengthening employability and social inclusion, and promoting environmental education and protection. Sustaining these benefits has become the main objectives of the social enterprise out of its sense of social and environmental responsibility (Fig. 3).

Based on the interview materials, i.e., perceptions and opinions expressed by the social enterprise staff and participants, the authors identified motivations and challenges that are particularly relevant to project delivery of the social enterprise in question, which are supported by direct quotes from the interviewees. All names have been changed to maintain anonymity.

Figure 3 depicts which forest ecosystem services are being exploited in the projects of the social enterprise, and in which form, their benefits are sustained through the projects and then delivered to the society. Enabling these benefits has become the objectives of the social enterprise.

### Motivations

#### Improving Well-Being Through Social Work Satisfaction

One of the central concerns inherent in social enterprises is to provide a means of working with individuals, communities, and the society they live in. Through active participation in the projects offered by the social enterprise, participants can not only develop skills and capacities to achieve their individual inspiration, but also gain more insights into their conditions by working together with people sharing similar health issues. Both of these two aspects can have a positive influence on their physical and psychological health. These activities give people recognition and satisfaction, and improve their mental conditions as one participant explained:

Physically the activities have made me realise that my arthritis and age are not really important […] achieving things helps, working with my hands helps […] and my self-esteem has grown. (Participant 4).

Notably, many of the participants experienced mental health issues. In response, the social enterprise provides work opportunities with low-entry thresholds particularly suitable for those experiencing mental hardship. It is known that health and wellbeing are closely related, not only to individual functioning, but also societal integration and participation (Baumgartner and Burns 2014). Through participation and interaction with others, these activities are expected to make the participants feel less marginalised and isolated, and thus improve their wellbeing.

In addition, the social enterprise uses forest ecosystem services to innovate projects that specifically target their participants. It provides ‘forest therapy’ with a variety of not only physical but also aesthetic activities that can relieve stress and facilitate mental relaxation. This is supported by a recent study that forests themselves can provide ‘green care’ that has a therapeutic effect on easing nerves, and may improve mental health conditions (Ohe et al. 2017).

As a result, forest ecosystem services not only provide an amenity where social connections can establish and grow, but also its innate therapeutic effects. Both of these factors may contribute to the wellbeing of the participants. In this respect, social enterprises may have the potential to play an important role in tackling health inequality and restructuring public health services in Scotland through forest-based practices.

#### Increasing Employability and Encouraging Employment

A second major motivation of the social enterprise is to help disadvantaged people who are at the risk of exclusion from the job market. The social enterprise devised a suite of activities under its ‘people’ project specially designed for the participants with regard to their circumstances and sensibilities. Depending on the capacity of individual participants, they can start from basic forest-related skills such as bush clearing and firewood collecting. As their confidence grows, participants are encouraged to undertake work of various levels of difficulty such as woodcraft and tree thinning with appropriate tools in woodlands. These teaching activities are often supported by the provisioning services of forest ecosystems, ranging from carving wooden products, making juices, jams and chutneys, building rustic furniture, to seasonally planting trees.

During the field observation, it was noted that these learning programmes offered by the social enterprise aim to train participants to find their own working pace and to optimise the workload without feeling pressured. By teaching participants land-based skills and making eco-friendly quality products, the social enterprise expects to improve their employability through exercises of manual forest-based practices. In addition, participants can make their own decisions as to what products to make according to their hobbies and interests at a later stage when they become skilled, giving them the opportunity to feel the power of decision-making. Their products are often given to people as gifts, kept as personal souvenirs, and most importantly, sold as a form of self-employment. Participants seem to acknowledge the benefit of these activities on their relation to the job market, as one participant stated:

Especially when I was going through hard times […] (the social enterprise) showing me that I can do things if I put my mind to it and showing me the right path to take with my new career as well as my future. (Participant 7).

The social enterprise therefore mimics many facets of a real-world workplace environment, in which participants can be seen as temporarily employed. This finding is in line with a previous study that social enterprises play an important role in providing workplaces suffused with social supports to enhance the employability and stimulate work attachment of participants (Chan 2016). Participants are willing to demonstrate their work and help each other as colleagues, which not only helps build up their social relations, but also exercises their communication and collaboration skills regularly. This may further enhance participants’ sense of self-worth within the work context and help sustain their engagement. As a result, their employability is expected to increase, and the positive benefits brought by being employed—though not officially—on the wellbeing of people are expected to achieve. Participants receive the benefits of employment, mostly in the form of individual recognition and sometimes financial reward generated from selling their products. The symbolism of being rewarded in either form exerts a powerful influence on participants’ wellbeing (Macaulay et al. 2018).

It should be stressed that forest ecosystem services cannot directly produce these multiple benefits. But through forest-based activities, the social enterprise enhances people’s access to forest ecosystem services and safeguards the continuity of its provision. Participants thus can receive various benefits during the process, and some subsidiary benefits may also flow to a wider community such as participants’ families (Farmer et al. 2012).

#### Promoting Environmental Education to the Public

Last but not least, the social enterprise attempts to provide wide access to environmental education to the public and recall people’s interests in forestry via its ‘planet’ project. The project contains a suite of activities that are made available to the public, including forest visits, woodland management activities and workshops on forest-related skills. The main objective is to strengthen people’s connection with nature, in particular forests, and consequently shift their attitudes and behaviours via incidental learning opportunities on environmental practices, as the former manager stressed:

For us, the motivation was initially to build a strong woodland culture […] make more native species woodlands in Scotland […] for Scottish people to become more aware of climate change and global biodiversity loss. (Former manager).

A recent survey in the UK states that one third of the UK public is reluctant to adapt their lifestyles in an environmentally-friendly way, especially the older generations (Natural England 2018). The social enterprise shares this concern and uses forests as innovative ‘outdoor classrooms’ where environmental education can be promoted to the public. The efficacy of outdoor learning experiences is recorded as being better than classroom-based learning, indicated by improved memories and scores and particularly changed attitudes and behaviours (Dillon et al. 2006). This form of education may also be useful for those who feel excluded from mainstream education, usually those who are in disadvantageous socio-economic positions. It is observed that these educational activities are based on small-scale forestry practices, such as seasonal planting and thinning, and the number of participants is limited on each event. In this way, the social enterprise expects to improve the quality of the education and deliver effective results of the shift in environmental attitudes, values and behaviours.

In addition, the state of forestry education in the UK has attracted widespread concerns due to the considerable decline of student interest and enrolment (Leslie et al. 2006). In response, the social enterprise has expressed the aim of stimulating public interest in this traditional subject and building a strong woodland culture. By providing access to managing forests and encouraging participants to conduct small-scale forestry practices, the social enterprise hopes to recall people’s passion for traditional subjects that are related to the environments. It also acts as a reminder to preserve traditional manual skills by teaching participants to use appropriate tools to manage forests sustainably.

These activities are supported by the cultural services of forest ecosystems, i.e., activities in and interactions with forests. The social enterprise hopes that by sustaining activities that increase people’s connection with nature and activities that improve their environmental education as well as awareness, long-term impacts brought by their attitudinal and behavioural change can be achieved. As the interviewee stressed:

My view is that a strong and effective woodland culture can have a much bigger impact and that it can play a significant role in tackling climate change, which is a global challenge that will play out over centuries. (Practitioner 2).

### Challenges

Despite the opportunity to spread diverse benefits derived from forest ecosystems, the social enterprise faced several challenges imposed by both internal and external factors.

#### Internal Issues Faced by Staff Members and Management

Staff members with appropriate skills and experience are crucial for an organisation to function and thrive. Through observation, it is noticed that social enterprises, especially those concerning themselves with forest-related issues, must be equipped with high levels of professional knowledge and technical skills. More importantly, they also need sophisticated people skills, as one interviewee said:

I think you have to have people skills […] the skills of working for a social enterprise is a combination of technical, or professional skills if you like, and also people skills. (Practitioner 1).

The staff members in this case often introduce forest-related knowledge before practice and strive to establish strong bonds between participants and the environment. These bonds, in turn, create a close and friendly co-working environment where the provision of high-quality forest ecosystem services can be facilitated. Therefore, staff members are of great importance in organisational efficiency and ecosystem services delivery, which makes recruiting suitable candidate highly desirable but challenging, not to mention appointing an appropriate manager.

Previous research has also reported that employing appropriate members has become increasingly difficult in the third sector, to which social enterprises belong (Spear et al. 2009). Although the Scottish Government provides a series of entrepreneurial learning programmes, training workshops and networking opportunities across the sector, they are not fully embraced by the social enterprise in question. The main reasons are that these activities appear rather general and not enough locally ‘tailored’ to match the needs of the social enterprise, and that they are often in clash with other managerial responsibilities, as the interviewees explained:

There is a lot of freely available training at a very basic level, less as your needs become more complex. (Practitioner 5).

We have no spare capacity to engage with these activities […] We could make time but that would come at the expense of our income generation or at some other aspects of management. (Current manager).

At the time of the interviews, the social enterprise was experiencing a critical time of financial difficulty. This may imply that economic elements, such as fundraising and generating income, may take precedence when income generation is not secured. Whereas the social and environmental benefits are usually regarded as the immediate priority when the social enterprise operates smoothly. Unlike other types of organisations such as charity and business enterprise, social enterprises often conduct business on a multiple-objective basis. It is the manager who is responsible for considering the multiple interests of different stakeholders (such as participants, staff members, and funders) and balancing the social, economic and environmental objectives of the social enterprise. It has been highlighted that the stakeholder landscape of forestry is particularly complex in the UK given the breadth of interests of a diverse range of stakeholders which spans from the local to the national scale (Raum 2018). Therefore, this issue of reconciling multiple needs is particularly exacerbated when forest ecosystem services is involved as the outcomes are often perceived and evaluated differently by different stakeholders.

#### External Challenges Imposed by the Current Funding Norms

Social enterprises’ financial security is often safeguarded by revenues coming from grants and matched funding as well as some self-generated interests. Government funding, which is often ample if applied successfully, can have a strong effect on the development of social enterprises, as the interviewee said:

New Caledonian Woodlands does not receive any funding from government sources and that makes the funding situation quite difficult […] these (funding challenges) are the things that cause the organisation to crumble down very quickly because they have no security of funding base. (Current Manager).

However, many small-sized social enterprises often have no competitive strength to obtain this privilege, and this is often the case of the social enterprise under investigation. The definition of social enterprise may have led to this issue but, more importantly, the current funding policy may have failed to take a comprehensive look at their outcomes, as the interviewee suggested:

It (a governmental organisation) struggles with the concept of social and environmental enterprise so sometimes […] The forest stewardship and forest ecosystem service aspects of our work which we enable to happen are not counted as part of our outputs (Former manager).

Previous literature has also identified social enterprises as organisations that are at a significant level of financial risk (Defourny and Nyssens 2010). It is particularly challenging for forest-based social enterprises because many forest ecosystem services are immeasurable and of long-term effect. Many interviewees expressed this concern as it is often ignored by funders’ current aspirations. Interviewees also emphasised that the current funding model is “unsustainable” as most funding runs every single year.

In reaction to insufficient grant funding, social enterprises sometimes become more business-like, creating self-generated revenues to support their social and environmental missions. Recent literature, however, has expressed the concern of social enterprises becoming too focused on their trading activities which may have a negative impact on their social objectives (Doherty et al. 2014). In addition, it is also worried that trading forest ecosystem services may negatively impact forest health (Ambrose-Oji et al. 2015). In the case of New Caledonian Woodlands, a considerable proportion of revenue is contributed by trading forest products provided the provisioning ecosystem services. It is indicated that the delivery of forest ecosystem services could be facilitated with trading income, as the interviewee said:

(The economic benefit) is not the priority but important. It is necessary but without that earning, we will still seek, trying to make that project possible. But it becomes more feasible with income. (Current manager).

## Discussion

By synthesising the primary data collected from the case study in Scotland and the secondary data drawn from existing literature and UK/Scottish Government policy documents, this paper summarises the motivations and challenges of forest-based social enterprises in delivering forest ecosystem services. Based on the results, this paper attempts to flag two priorities that may close the gap between knowledge and policy implementation, targeted at the concepts of ecosystem services and social enterprises respectively.

### Encourage Ecosystem Services Based Tools for Decision-Making

The findings suggest that a large part of the benefits delivered by the forest-based social enterprise come under the category of cultural ecosystem service, such as improving participants’ wellbeing and promoting environmental education. Many of these benefits come from small-scale forest practices and are likely to exert long-term social and environmental impacts. However, current institutional frameworks are still insufficient in assessing these intangible values, and thus are left neglected or ignored in real-world practices. This echoes the finding that many ecosystem services remain to be captured and assessed, particularly those cultural services (Ludwig 2000) as well as social values ascribed to provisioning, regulating and supporting services (Plieninger et al. 2013). In addition, it is reported that limited efforts have been made to assess ecosystem services on small-scale operations due to the impact of larger scales that are difficult to count into (van Oort et al. 2015). In the case of the forest social enterprise under investigation, these two factors have imposed strong restrictions on the demonstration of their performance and efficiency, which has consequently placed it in a disadvantageous position.

Conversely, a practical approach to assessing and evaluating cultural ecosystem services may potentially offer a solution to resolve some of the challenges faced by the social enterprise. Primarily, it could resolve the challenge of balancing social, economic and environmental priorities and facilitate decision-making as ecosystem services initially set out to be. For example, it could help the social enterprise to add weight to individual stakeholders’ interests and consider multiple aspects before decision-making. Similarly, stakeholders could also use ecosystem services as a decision-making tool when a wide range of social, economic and environmental interests is involved. Secondly, to some extent it would tackle the funding challenge. With cultural ecosystem services valorised, there would be a benchmark against which the social enterprise could better demonstrate its long-term social and cultural benefits. Subsequently, it could be more realistic for the social enterprise to convince potential benefactors to provide funding.

However, new issues arise with the monetisation of cultural ecosystem services, regarding their possibility, difficulty and uncertainty. Firstly, whether or not social and cultural values can be valorised is a highly contentious question. Kumar and Kumar (2008) argued that the socio-cultural values of an individual’s relationship with nature cannot be expressed in monetary terms, because it neglects the value of psychological wellbeing stemming from that relationship. This is particularly so in the case of the social enterprise under investigation, because, according to the participants, the improvement in their sense of wellbeing derives not only from the amenity provided by forests, but also from the social teamwork and personal relationships built up during their activities. Secondly, even if it were possible, monetising ecosystem services remains difficult. The value of cultural ecosystem services cannot easily be captured with price tags, considering the wide range of services involved and how some of them are social constructs with little dependence of the state of ecosystems (Daniel et al. 2012). Nevertheless, ecosystem services-based decision-making tools provide a new perspective of tackling the challenges faced by those social enterprises that operate on natural ecosystems.

### Innovate Funding Mechanism for Social Enterprises

The results highlight a mismatch between funders’ aspirations and receivers’ needs regarding what a project involves and how long it should be funded. The interviewees suggested that the current funding tradition has an emphasis on social outcomes. This inclines the forest-based social enterprise to drift towards the social end of its projects, leaving the forest ecosystem services aspect uncounted when giving credits to their outputs. As a result, only the social side of the performance is acknowledged and funded while their contribution to forest stewardship and ecosystem services is largely neglected.

In addition, the current funding mechanism falls short to consider the time scale on which individual social enterprises operate. Social enterprises operating business in commercialised industries may not require long-term grant support because of their self-generated interests. However, in the case under investigation, the social enterprise’s projects are based on forest ecosystem services and may arguably never become commercially viable without external support (but they are likely to be socially and environmentally necessary). Therefore, the current funding mechanism has not fully recognised the importance of these two issues when finalising grant decisions on social enterprises.

Furthermore, social enterprises have only limited power to counterbalance their funders and the traditional reporting scheme further hinders them from demonstrating their performance. Funding given to the case under investigation is often associated with the overall aims and objectives that funders endeavour to achieve. In return, funders require the receiver to provide a report in order to help them measure the funding impact. However, the impact measured in the report is usually quantity-based, such as the number of participants attending events, the amount of days of events organised, and the volume of timber produced and traded. It seeks little in the way of detail concerning the quality of the projects.

Gordon et al. (2018) also noted that some social enterprises believed that their reporting methods fail to fully capture their project impacts. Although some social enterprises succeeded in gaining more recognition from traditional funders by collecting some additional ‘beautiful’ data (such as films and qualitative cases) to complement their traditional reports, they were not able to challenge the current funding discourse and structure. These efforts may contribute to the revision of particular funding rules and assessments, but only rarely are social enterprises able to achieve a profile sufficiently influential to harness the UK/Scottish policy environment, in which fundamental issues pertaining to social enterprises are nurtured and developed (Steinerowski and Steinerowska-Streb 2012).

Given the diversity of all types of social enterprises and of the fields in which they operate, it is necessary for governments and funders to reconsider and innovate their ‘traditional’ strategies and funding mechanisms. For example, large funding packages may be particularly applied to small social enterprises rather than those large ones often with strong enterprising functions. Since social enterprises are not yet prominent in the UK/Scottish policy setting, long-term funding is arguably a more suitable route to safeguarding their development.

## Conclusions

This paper explored the current UK/Scottish policy addressing ecosystem services and social enterprises and their motivations and challenges in sustaining forest ecosystem services, drawing on a case study in Scotland. Many of the objectives of the social enterprise are in line with the philosophy of forest ecosystem services, chiefly but not exclusively with respect to the wellbeing of people and the sustainability of the natural environment.

Through small-scale forest-related practices, the social enterprise has enabled many types of forest ecosystem services to benefit local society, and many of the benefits are under the provisioning and especially cultural categories of ecosystem services. Its motivations include improving people’s mental health conditions and employability as short-term objectives, as well as promoting public education and a green lifestyle in order to achieve long-term environmental impact. At the same time, they also face both internal and external challenges, ranging from recruiting appropriate members and managers to balancing the multiple objectives of the social enterprise. Financial security is often the external factor that causes social enterprises to crumble, and current funding and reporting mechanisms may have fallen short of fully assessing their performance, with long-term social and environmental effect largely neglected.

Currently, the lack of appropriate assessments of cultural ecosystem services hampers forest-based social enterprises from demonstrating their performance and impact, leaving them in a disadvantageous position in relation to the traditional funding norms. The ecosystem services-based thinking is currently missing in this field and a practical method of evaluating cultural services could potentially revolve some fundamental and commonly seen challenges faced by social enterprises that operate on small-scale forest-related practices.

Lastly, clear gaps exist between policy frameworks and their implementation. In Scotland, government strategies have aimed at safeguarding both forestry and social enterprises individually, but only few connections exist during their implementation processes. Forest-based social enterprises can play an important role in filling policy gaps and creating synergies between seemingly disconnected policies addressing ecosystem services and social enterprises. Due to the small sample size, the findings of this paper are necessarily suggestive rather than conclusive. Further research should be encouraged on social enterprises of the same kind as the one cited here, as well as those that operate in other specific ecosystems.
